# Bridging the awareness-action gap in pelvic floor prehabilitation: cognitive barriers and facilitators for asymptomatic populations—a perspective

**DOI:** 10.3389/fpubh.2026.1700346

**Published:** 2026-02-13

**Authors:** Chun-hua Wu, Xiao-xiao Huang, Ling-mei Tan, Wei-hong Chen, Li Zheng, Zhe-cheng Zeng

**Affiliations:** 1 Department of Gynecology, Nanfang Hospital, Southern Medical University, Guangzhou, China; 2 Department of Urological Surgery, Nanfang Hospital, Southern Medical University, Guangzhou, China; 3 Reproductive Medicine Center, Nanfang Hospital, Southern Medical University, Guangzhou, China

**Keywords:** asymptomatic population, cognitive barriers, pelvic floor disorder, perspective, prehabilitation, urinary incontinence

## Abstract

Pelvic floor disorders (PFDs), including urinary incontinence and pelvic organ prolapse, represent a widespread public health concern with substantial implications for functional status and quality of life. Evidence supports the efficacy of prehabilitation—a proactive strategy focused on preventing dysfunction before clinical onset—through screening and early intervention. However, its implementation in asymptomatic populations remains limited, largely due to a pervasive “awareness-action gap” wherein knowledge fails to translate into behavioral engagement. This perspective article systematically examines the cognitive, psychosocial, and structural determinants that act as barriers or facilitators to participation in pelvic floor health initiatives. By integrating the Health Belief Model and Nudge Theory, this study investigates factors influencing health engagement. The framework identifies major barriers, including asymptomatic complacency, knowledge gaps, low self-efficacy, and systemic obstacles. It also highlights potent facilitators, such as targeted message framing, cognitive schema alignment, credible messengers, and purposeful choice architecture. We further propose a multilevel framework for bridging this gap, combining targeted health communication, clinical integration of preventive protocols, digital health tools, and supportive policy reforms. Ultimately, transforming awareness into sustained action will require a coordinated effort across health systems, incorporating evidence-based behavioral interventions and aligning incentives to establish pelvic health promotion as a public health priority.

## 1 Introduction

### 1.1 The burden of pelvic floor disorders

Pelvic floor disorders (PFDs), encompassing conditions such as urinary incontinence and pelvic organ prolapse, constitute a widespread but frequently overlooked public health issue (1, 2). Epidemiological research reveals that a large percentage of women will develop at least one form of PFDs during their lifetime, with prevalence rates positively correlated with factors such as age, parity, and body mass index (1, 3). These disorders significantly impair physical function, mental health, social interactions, and overall quality of life, in addition to generating substantial economic costs for healthcare systems and affected individuals (4, 5). Despite their prevalence and impact, PFDs often go unreported and untreated, partly due to perceptions that they are an unavoidable aspect of aging or childbirth, thereby contributing to their status as a “silent epidemic.”

Beyond their clinical impact, pelvic floor disorders also generate significant economic and social burdens. Direct medical costs stem from prolonged conservative treatments—such as multiple physiotherapy visits, continence aids, and medications—and from surgical procedures like midurethral slings and pelvic organ prolapse repairs, all of which involve considerable expenses (6, 7). These are compounded by indirect societal costs, including lost workplace productivity, caregiver burdens, and negative psychological effects like anxiety and social isolation (8–10). The substantial scale of these combined burdens argues for the cost-effectiveness of prevention (7). Therefore, enhancing engagement in pelvic floor prehabilitation could lower future healthcare demands and lessen the wider societal consequences of these disorders.

### 1.2 From treatment to prevention: the rise of prehabilitation

In light of these challenges, a transition from reactive treatment to proactive prevention has begun to influence contemporary healthcare practice. Central to this shift is the concept of prehabilitation—a preventive strategy aimed at identifying risk factors and implementing early interventions before clinical symptoms manifest (11, 12). Within pelvic health, prehabilitation includes systematic screening, patient education on pelvic floor physiology, and guided preventive exercises such as pelvic floor muscle training (13, 14). Growing evidence indicates that such proactive measures, especially among high-risk groups including pregnant and postpartum women, can decrease the incidence and severity of subsequent PFDs (13, 15). Thus, prehabilitation represents not only a clinical alternative but also a necessary component of sustainable healthcare.

### 1.3 Defining the “Awareness-Action Gap”

Nevertheless, the integration of these evidence-based approaches into routine practice remains limited. A major impediment is the significant disconnect between acknowledged benefits of prehabilitation and actual participation among asymptomatic individuals (16, 17). This discrepancy, referred to here as the “Awareness-Action Gap,” highlights the divide between acquired knowledge of preventive benefits and the tangible adoption of health-promoting behaviors (17). This gap suggests that awareness alone is inadequate to prompt behavioral change; rather, it is influenced by a complex interplay of cognitive, psychosocial, and structural determinants. This perspective article aims to dissect the cognitive underpinnings of this gap, analyzing both barriers to and facilitators of behavioral change, and to propose an integrated strategy for promoting prehabilitation in asymptomatic populations.

## 2 Deconstructing the cognitive barriers

### 2.1 Lack of salience and asymptomatic complacency

The limited uptake of pelvic floor prehabilitation among asymptomatic individuals stems from a complex array of cognitive, psychological, and structural barriers that collectively inhibit proactive engagement. A fundamental obstacle is the inherent lack of perceptual salience and the consequent asymptomatic complacency (18). In the absence of overt physical symptoms, pelvic floor health fails to register as an immediate concern within an individual’s health priorities (19). This absence of tangible threat suppresses perceived susceptibility—a core component of Health Belief Model (HBM)—fostering a state of complacency characterized by beliefs such as “this does not apply to me” or “I am not at risk.” Consequently, preventive action is perpetually deferred in favor of more salient health issues.

### 2.2 Knowledge deficits and misconceptions

Further exacerbating this inertia are substantial knowledge gaps and common misconceptions. Many individuals lack basic awareness of pelvic floor anatomy, its role in urinary, bowel, and sexual function, and modifiable versus non-modifiable risk factors—including vaginal delivery, chronic intra-abdominal pressure, and hormonal changes (20, 21). Equally detrimental are pervasive sociocultural misbeliefs that frame pelvic floor disorders as an unavoidable part of aging or a taboo subject undeserving of open discourse (22). Such stigmatization not constrains information-seeking but also discourages clinical consultation, thereby perpetuating a cycle of silence and inaction.

### 2.3 Psychological and perceptual hurdles

Deeper cognitive and affective processes also play a decisive role. Optimism bias leads individuals to systematically underestimate their personal risk relative to others, creating a false sense of invulnerability. Meanwhile, low self-efficacy—often arising from uncertainty about performing exercises correctly or doubts regarding long-term adherence—undermines motivation to initiate or maintain prehabilitative routines (23, 24). The internal and non-visible nature of pelvic floor exercises, lacking external feedback or visible reinforcement, frequently intensifies these confidence-related barriers (24).

### 2.4 System-level and environmental barriers

Ultimately, these individual-level cognitive barriers are compounded by systemic and environmental impediments. A pronounced clinical practice gap is evident; many healthcare providers lack the time, training, or institutional incentives to incorporate preventive pelvic health counseling into standard care (25). The scarcity of structured screening programs and financial reimbursement for prehabilitation consults further signals its low priority within healthcare systems (25). Moreover, practical constraints related to the accessibility and acceptability of services—such as geographic availability, cost, and apprehensions about discomfort or embarrassment during clinical evaluations—deter even well-informed individuals from seeking care (26). Thus, effectively addressing inaction requires a integrated approach that simultaneously targets individual cognition and the broader structural context.

### 2.5 Theoretical underpinning: the HBM as an explanatory framework

The cognitive barriers previously described can be effectively analyzed through the HBM (18), a theoretical framework widely used to understand health-related behaviors. According to the HBM, an individual’s likelihood of adopting preventive health measures depends on several key constructs: perceived susceptibility (personal risk of developing a condition), perceived severity (seriousness of the condition and its consequences), perceived benefits (effectiveness of the recommended action), perceived barriers (tangible and psychological costs of the action), and self-efficacy (confidence in performing the behavior) (27, 28). In pelvic floor prehabilitation, low salience and asymptomatic complacency reflect deficits in perceived susceptibility and severity (29). Knowledge gaps and misconceptions further distort perceptions of risk, severity, and potential benefits. Finally, low self-efficacy and psychological obstacles correspond directly to the HBM’s self-efficacy and perceived barriers constructs (28, 30). Thus, the HBM offers a coherent explanation for the gap between awareness and action, emphasizing that effective interventions must address this comprehensive set of beliefs to promote behavioral change.

## 3 Identifying cognitive facilitators: levers for change

Bridging the gap between awareness and action in pelvic floor prehabilitation requires a deliberate focus on cognitive and behavioral facilitators. Evidence from health psychology and behavioral economics suggests that targeted strategies can effectively promote engagement by aligning with how individuals process information and make decisions.

### 3.1 Strategic message framing: emphasizing empowerment and benefits

The effectiveness of communication about prehabilitation depends critically on how messages are constructed. Threat-based messages that focus exclusively on potential dysfunction may increase awareness but often elicit psychological reactance or avoidance (31, 32). In contrast, gain-framed messages that highlight positive outcomes and self-improvement tend to be more motivating for preventive behaviors (33). Emphasizing benefits such as “improved core stability,” “enhanced quality of life,” “maintenance of long-term functional independence,” and “investment in future health” aligns with values of autonomy and self-efficacy. This positive framing helps individuals reconceptualize prehabilitation not as a medical obligation, but as a voluntary pursuit of personal wellness and bodily integrity.

### 3.2 Cognitive schema alignment: integrating new information with existing knowledge

The assimilation of health information is facilitated when it connects to pre-existing cognitive frameworks (34). Concepts such as “core strength,” “preventive health,” “postnatal recovery,” and “healthy aging” are already familiar to many individuals. By explicitly linking pelvic floor health to these established cognitive schemas, educators and clinicians can reduce the cognitive effort required to comprehend its importance (35). For example, integrating the pelvic floor into discussions about overall core musculature and functional stability provides a relatable and biomechanically coherent context. This approach not only enhances comprehension but also increases the perceived relevance and personal applicability of preventive measures.

### 3.3 Credible messengers and social influence: building trust and normalizing behavior

The perceived credibility of the information source significantly affects its acceptance. Recommendations from trusted professionals—including primary care providers, gynecologists, midwives, and physiotherapists—lend authority and legitimacy to prehabilitation advice (36, 37). Beyond expert endorsement, social normative influence plays a powerful role in behavior change. Testimonials from peers, community-based group sessions, and visible participation by role models can effectively normalize prehabilitation, reduce perceived stigma, and generate positive social pressure (38, 39). This combination of expert validation and peer demonstration creates a supportive ecosystem that encourages both initiation and adherence.

### 3.4 Nudge-based strategies: architecting choices for better decisions

Insights from behavioral economics provide a robust framework for promoting health behaviors through subtle modifications to the choice environment. Nudge theory, which emphasizes the design of decision-making contexts without restricting options or employing coercive incentives, offers particularly valuable strategies for increasing participation in pelvic floor prehabilitation (40, 41). Effective interventions include the use of default options, such as automatically incorporating pelvic health screenings and referrals into standard care pathways for postnatal or menopausal patients, thereby leveraging the tendency to adhere to pre-set selections (40). The strategic communication of social norms—informally highlighting the growing prevalence of peer engagement in preventive practices—can reduce perceived stigma and generate positive social pressure. Furthermore, immediate feedback tools, including biofeedback devices and mobile health applications, offer real-time validation of correct technique, which enhances self-efficacy and reinforces consistent practice through operant conditioning (41, 42). Finally, facilitating the formation of implementation intentions by guiding individuals to concretely plan the timing, location, and method of exercises helps bridge the intention-behavior gap by linking goals to specific situational cues (42). Collectively, these approaches function by aligning the decision architecture with cognitive heuristics, reducing procedural barriers, and making the desired behavior—engagement in prehabilitation—the path of least resistance.

### 3.5 Synthesizing a theoretical framework: integrating HBM and nudge theory

The facilitators outlined in this section can be effectively operationalized through an integrated theoretical approach combining the HBM with Nudge Theory. While HBM provides a diagnostic framework for understanding why the awareness-action gap persists, Nudge Theory offers complementary mechanisms for bridging this gap through choice architecture that aligns with human cognitive tendencies (43). For example, strategically framed messages target HBM’s perceived benefits and barriers by emphasizing positive health gains and personal agency (43). Aligning information with existing cognitive schemas reduces cognitive effort—a significant barrier—by enhancing information accessibility (44). The deployment of credible messengers strengthens the perceived validity of information regarding susceptibility, severity, and benefits (44). Furthermore, specific nudges directly address HBM constructs: default options mitigate inertia and decision fatigue (addressing perceived barriers), social norms communication elevates perceived susceptibility and reduces stigma, and real-time feedback mechanisms enhance self-efficacy (43, 45). Together, the integration of HBM and Nudge Theory offers a theoretically grounded, multi-level framework for designing interventions that effectively translate awareness into sustained preventive health behavior.

**Figure 1:**
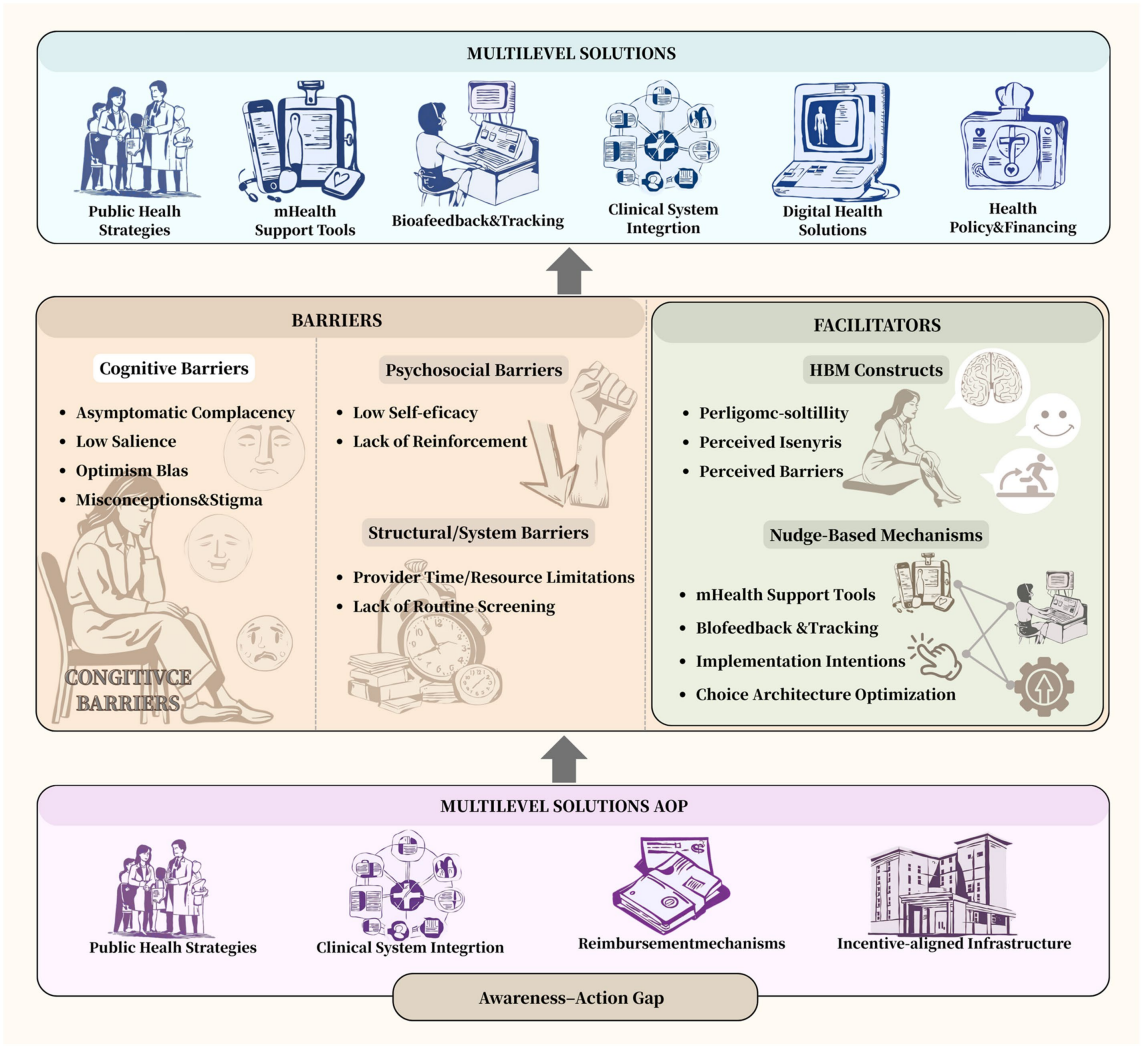
Conceptual model of the awareness-action gap and multilevel strategies to promote pelvic floor prehabilitation. Created with BioRender.com.

To offer an integrated understanding of the interactions among barriers, facilitators, and multi-level strategies, we have introduced a conceptual model (Figure 1). This model places the “awareness–action gap” at its core and outlines the cognitive, psychosocial, and structural determinants that hinder action. It then integrates evidence-based facilitators informed by the Health Belief Model and Nudge Theory, showing how these can be strategically applied to address key barriers. Finally, the model connects these determinants to potential solutions at the public health, clinical, digital health, and policy levels. This provides a comprehensive and logically structured visualization of the manuscript’s theoretical framework.

## 4 A multifaceted blueprint for bridging the gap

Addressing the awareness-action gap in pelvic floor prehabilitation necessitates an integrated, multi-system approach that aligns interventions across public, clinical, technological, and policy dimensions. Sustainable change requires coordinated action that recognizes the interplay between individual motivation, healthcare delivery structures, and broader socioeconomic incentives.

### 4.1 Public health strategy: destigmatization and targeted education

National and regional health authorities should initiate evidence-based public health campaigns aimed at normalizing pelvic health discourse and elevating its priority within preventive care (46, 47). These initiatives can draw upon successful models from other health awareness domains, utilizing mass media channels alongside digitally targeted outreach to specific at-risk demographics (e.g., parous women, aging populations) (46, 47). Messaging must be developed through participatory design with target communities to ensure cultural relevance and psychological acceptability (48). The objective is to foster an environment where pelvic health maintenance is regarded as an integral component of overall health self-management, thereby reducing secrecy and shame.

### 4.2 Clinical system integration: standardizing preventive care pathways

Healthcare institutions play a critical role in operationalizing prehabilitation through systematic clinical practice change. This requires dual strategies: first, enhancing provider capacity through specialized training in preventive pelvic health communication and counseling techniques (49); second, restructuring clinical workflows to embed prevention into routine care (36). Practical steps include the adoption of standardized risk-assessment tools during prenatal visits, postpartum evaluations, and midlife health screenings (50). Electronic health record systems can be leveraged to prompt clinicians, document discussions, and facilitate referrals. Such integration ensures that pelvic health becomes a consistent element of preventive medicine rather than a niche or reactive service (51).

### 4.3 Digital health solutions: supporting adherence and self-management

Technology-mediated interventions present scalable opportunities to support behavior change and maintain long-term engagement. Digital platforms, including mobile health applications and remote monitoring technologies, should offer structured educational content, personalized exercise programs, and adherence reminders (52). Incorporating interactive components such as video demonstrations of correct technique and progress tracking enhances self-efficacy (53). More advanced systems may integrate sensor-based biofeedback to provide objective performance measures, creating a closed-loop system that reinforces correct practice and sustains motivation through visible progress indicators (53).

### 4.4 Health policy and financing: creating enabling structural conditions

Long-term sustainability depends on creating supportive policy and financing environments. Key priorities include securing reimbursement for evidence-based pelvic health prehabilitation services through public and private insurance schemes, which reduces financial barriers for patients and incentivizes provider delivery (12). Simultaneously, policy makers should consider incorporating quality metrics related to preventive pelvic care into value-based payment programs (54). Advocacy efforts must emphasize the cost-effectiveness of prevention relative to long-term treatment of pelvic floor disorders, aligning economic incentives with public health goals and encouraging institutional investment in preventive services (55). The economic burden of these disorders highlights the urgency of addressing the awareness–action gap. The lack of early preventive action contributes to rising long-term treatment costs for individuals and healthcare systems.

### 4.5 Implementation roadmap and strategic priorities for resource-limited settings

A structured and pragmatic roadmap is essential for translating the multilevel blueprint into practice, particularly in resource-limited settings. Prioritization should begin with interventions that are feasible, low-cost, and capable of producing meaningful early gains (56, 57). In the short term, integrating basic preventive measures into routine clinical care represents the most achievable starting point. Standardized risk screening during prenatal, postpartum, and midlife visits, together with brief and structured counseling supported by electronic prompts or simple educational tools, can be incorporated with minimal additional resources (58). Low-cost digital supports—such as text-based reminders or short instructional videos—further reinforce adherence and promote self-management (59).

As foundational clinical practices become established, medium-term efforts should focus on expanding digital and community-based support systems to increase reach and continuity of care (49). Enhancing mobile health platforms with personalized exercise guidance, progress tracking, and optional low-cost biofeedback functions can strengthen engagement and reliability of performance (60). Community-based education programs delivered through primary care networks, maternity services, or local organizations help normalize pelvic health conversations and reduce stigma (61). Social-norm strategies that highlight peer participation and collective benefits can further enhance motivation and acceptance.

Long-term sustainability requires alignment with broader policy and financing structures. Establishing reimbursement pathways for evidence-based prehabilitation services lowers financial barriers and encourages provider participation (62). Incorporating preventive pelvic-health indicators into national quality metrics reinforces accountability and institutional commitment (62). Public health campaigns implemented at regional or national levels can further destigmatize pelvic floor health and elevate its priority within preventive care (63).

To ensure synergy across these sequential phases, coordination mechanisms are essential. Digital tools should support and extend clinical counseling, while policy incentives should reinforce standardized care pathways and community engagement. Linking community education programs with clinical referral systems helps maintain continuity and ensures that preventive actions are embedded within the broader health system. Through this phased and interconnected approach, the roadmap provides a realistic path for initiating, scaling, and sustaining pelvic floor prehabilitation across diverse resource settings.

## 5 Summary and future directions

In summary, addressing the cognitive and behavioral barriers that drive the awareness–action gap is essential to realizing the preventive value of pelvic floor prehabilitation. This study outlines major obstacles—including asymptomatic complacency, knowledge deficits, low self-efficacy, and system-level constraints—and identifies corresponding facilitators informed by the Health Belief Model and Nudge Theory. Translating awareness into sustained preventive behavior requires coordinated efforts across clinical practice, public health, behavioral science, and health policy. The proposed multilevel framework, together with the phased implementation roadmap, provides a structured strategy for integrating targeted communication, standardized clinical pathways, digital support tools, and enabling policy measures, with particular applicability in resource-limited settings. Future research should focus on developing and testing tailored interventions for diverse demographic and cultural groups, implementing theory-driven randomized trials to evaluate specific behavior change techniques, and conducting longitudinal studies to assess the long-term effectiveness of prehabilitation programs in real-world settings. To translate these insights into tangible health gains, pelvic floor health must be recognized as a preventable public health priority. Strategic investment in evidence-based prevention strategies, supported by aligned policy and financing, is essential to reduce the future incidence of pelvic floor disorders, alleviate associated personal suffering, and mitigate long-term healthcare costs.

## Data Availability

The original contributions presented in the study are included in the article/supplementary material, further inquiries can be directed to the corresponding author/s.

## References

[1] Kenne KA Wendt L Brooks Jackson J. Prevalence of pelvic floor disorders in adult women being seen in a primary care setting and associated risk factors. Sci Rep. (2022) 12:9878. doi: 10.1038/s41598-022-13501-w 35701486 PMC9198100

[2] Peinado Molina RA Hernández Martínez A Martínez Vázquez S Martínez Galiano JM. Influence of pelvic floor disorders on quality of life in women. Front Public Health. (2023) 11:1180907. doi: 10.3389/fpubh.2023.1180907 37942254 PMC10629477

[3] Peinado-Molina RA Hernández-Martínez A Martínez-Vázquez S Rodríguez- Almagro J Martínez-Galiano JM. Pelvic floor dysfunction: prevalence and associated factors. BMC Public Health. (2023) 23:2005. doi: 10.1186/s12889-023-16901-3 37838661 PMC10576367

[4] AlQuaiz AM Kazi A AlYousefi N Alwatban L AlHabib Y Turkistani I. Urinary incontinence affects the quality of life and increases psychological distress and low self-esteem. Healthcare (Basel). (2023) 11:1772. doi: 10.3390/healthcare11121772 37372891 PMC10297870

[5] Chen W Gong J Liu M Cai YC. Long-term health outcomes and quality of life in women with untreated pelvic floor dysfunction: a single-center cohort study. Front Public Health. (2025) 12:1495679. doi: 10.3389/fpubh.2024.1495679 39839434 PMC11746105

[6] Chong EC Khan AA Anger JT. The financial burden of stress urinary incontinence among women in the United States. Curr Urol Rep. (2011) 12:358–62. doi: 10.1007/s11934-011-0209-x, 21847532

[7] Coyne KS Wein A Nicholson S Kvasz M Chen CI Milsom I. Economic burden of urgency urinary incontinence in the United States: a systematic review. J Manag Care Pharm. (2014) 20:130–40. doi: 10.18553/jmcp.2014.20.2.130 24456314 PMC10437639

[8] Sung VW Washington B Raker CA. Costs of ambulatory care related to female pelvic floor disorders in the United States. Am J Obstet Gynecol. (2010) 202:483.e1–4. doi: 10.1016/j.ajog.2010.01.015, 20227673 PMC2866792

[9] Dakic J Perraton L Lindstrom J Hain E Chuah S Stay S. The hidden challenge: pelvic floor symptoms and their impact on performance and well-being in elite female Rugby players. Eur J Sport Sci. (2025) 25:e70013. doi: 10.1002/ejsc.70013, 40650437 PMC12254577

[10] Laurenzana L Fitzgerald C Bennis S. Pelvic pain and pelvic floor disorders in women: a physiatrist's approach to epidemiology and examination. Phys Med Rehabil Clin N Am. (2025) 36:311–28. doi: 10.1016/j.pmr.2024.11.008, 40210364

[11] Fleurent-Grégoire C Burgess N McIsaac DI Chevalier S Fiore JF Jr Carli F . Towards a common definition of surgical prehabilitation: a scoping review of randomised trials. Br J Anaesth. (2024) 133:305–15. doi: 10.1016/j.bja.2024.02.035 38677949 PMC11282475

[12] Driessens H Wijma AG Buis CI Nijkamp MW Nieuwenhuijs-Moeke GJ Klaase JM. Prehabilitation: tertiary prevention matters. Br J Surg. (2024) 111:znae028. doi: 10.1093/bjs/znae028 38436470 PMC10910596

[13] Romeikienė KE Bartkevičienė D. Pelvic-floor dysfunction prevention in prepartum and postpartum periods. Medicina (Kaunas). (2021) 57:387. doi: 10.3390/medicina57040387, 33923810 PMC8073097

[14] National Guideline Alliance (UK). Pelvic floor muscle training for the prevention of pelvic floor dysfunction: pelvic floor dysfunction: prevention and non-surgical management: evidence review F. London: National Institute for Health and Care Excellence (NICE) (2021). 35438876

[15] Ren S Gao Y Yang Z Li J Xuan R Liu J . The effect of pelvic floor muscle training on pelvic floor dysfunction in pregnant and postpartum women. Phys Act Health. (2020) 4:130–41. doi: 10.5334/paah.64

[16] Karlsson E Dahl O Rydwik E Nygren-Bonnier M Bergenmar M. Older patients' attitudes towards, and perceptions of, preoperative physical activity and exercise prior to colorectal cancer surgery-a gap between awareness and action. Support Care Cancer. (2020) 28:3945–53. doi: 10.1007/s00520-019-05237-7, 31863214 PMC7316666

[17] Conner M Norman P. Understanding the intention-behavior gap: the role of intention strength. Front Psychol. (2022) 13:923464. doi: 10.3389/fpsyg.2022.923464 35992469 PMC9386038

[18] Jones CL Jensen JD Scherr CL Brown NR Christy K Weaver J. The health belief model as an explanatory framework in communication research: exploring parallel, serial, and moderated mediation. Health Commun. (2015) 30:566–76. doi: 10.1080/10410236.2013.873363, 25010519 PMC4530978

[19] Sawettikamporn W Sarit-Apirak S Manonai J. Attitudes and barriers to pelvic floor muscle exercises of women with stress urinary incontinence. BMC Womens Health. (2022) 22:477. doi: 10.1186/s12905-022-02067-4, 36435776 PMC9701389

[20] Fante JF Silva TD Mateus-Vasconcelos ECL Ferreira CHJ Brito LGO. Do women have adequate knowledge about pelvic floor dysfunctions? A systematic review. Rev Bras Ginecol Obstet. (2019) 41:508–19. doi: 10.1055/s-0039-1695002, 31450258 PMC10316817

[21] Mckay ER Lundsberg LS Miller DT Draper A Chao J Yeh J . Knowledge of pelvic floor disorders in obstetrics. Female Pelvic Med Reconstr Surg. (2019) 25:419–25. doi: 10.1097/SPV.0000000000000604, 30074917

[22] Cox CK Schimpf MO Berger MB. Stigma associated with pelvic floor disorders. Female Pelvic Med Reconstr Surg. (2021) 27:e453–6. doi: 10.1097/SPV.0000000000000961 33105346

[23] Er-Rabiai Y Torres-Lacomba M Casaña J Núñez-Cortés R Calatayud J. Correlation of self-efficacy for pelvic floor muscle exercise with symptoms of stress urinary incontinence in women. Int Urogynecol J. (2024) 35:1487–93. doi: 10.1007/s00192-024-05818-z, 38861006

[24] Burkert S Knoll N Scholz U Roigas J Gralla O. Self-regulation following prostatectomy: phase-specific self-efficacy beliefs for pelvic-floor exercise. Br J Health Psychol. (2012) 17:273–93. doi: 10.1111/j.2044-8287.2011.02037.x, 22103706

[25] Zoorob D Higgins M Swan K Cummings J Dominguez S Carey E. Barriers to pelvic floor physical therapy regarding treatment of high-tone pelvic floor dysfunction. Female Pelvic Med Reconstr Surg. (2017) 23:444–8. doi: 10.1097/SPV.0000000000000401 28145917

[26] Hirschhorn AD Kolt GS Brooks AJ. Barriers and enablers to the provision and receipt of preoperative pelvic floor muscle training for men having radical prostatectomy: a qualitative study. BMC Health Serv Res. (2013) 13:305. doi: 10.1186/1472-6963-13-305 23938150 PMC3751161

[27] Rosenstock IM. The health belief model and preventive health behavior. Health Educ Monogr. (1974) 2:354–86. doi: 10.1177/109019817400200405

[28] Jones CJ Smith H Llewellyn C. Evaluating the effectiveness of health belief model interventions in improving adherence: a systematic review. Health Psychol Rev. (2014) 8:253–69. doi: 10.1080/17437199.2013.802623, 25053213

[29] Xu P Jin Y Guo P Xu X Wang X Zhang W . Barriers and enablers of pelvic floor rehabilitation behaviours in pregnant women with stress urinary incontinence: a qualitative analysis using the theoretical domains framework. BMC Pregnancy Childbirth. (2023) 23:300. doi: 10.1186/s12884-023-05633-2, 37118702 PMC10148524

[30] Yakıt Yeşilyurt S Yıldız ED İnal B Ayaz Taş S Çankaya H Başol Göksülük M . Investigation of pelvic floor knowledge, awareness and healthcare seeking in women with urinary incontinence: a cross-sectional study. Northwest Med J. (2024) 4:70–8. doi: 10.54307/2024.NWMJ.109

[31] Reynolds-Tylus T. Psychological reactance and persuasive health communication: a review of the literature. Front Commun. (2019) 4:56. doi: 10.3389/fcomm.2019.00056

[32] Hall MG Sheeran P Noar SM Ribisl KM Bach LE Brewer NT. Reactance to health warnings scale: development and validation. Ann Behav Med. (2016) 50:736–50. doi: 10.1007/s12160-016-9799-3 27333895 PMC5055422

[33] Mikels JA Young NA Liu X Stine-Morrow EAL. Getting to the heart of the matter in later life: the central role of affect in health message framing. Gerontologist. (2021) 61:756–62. doi: 10.1093/geront/gnaa128, 32915207 PMC8276605

[34] Nipa SI Sriboonreung T Paungmali A Phongnarisorn C. The effects of pelvic floor muscle exercise combined with Core stability exercise on women with stress urinary incontinence following the treatment of nonspecific chronic Low Back pain. Adv Urol. (2022) 2022:2051374. doi: 10.1155/2022/2051374 36105867 PMC9467742

[35] Crawford T Roger P Candlin S. Supporting patient education using schema theory: a discourse analysis. Collegian. (2018) 25:501–7. doi: 10.1016/j.colegn.2017.12.004

[36] Grant A Currie S. Qualitative exploration of the acceptability of a postnatal pelvic floor muscle training intervention to prevent urinary incontinence. BMC Womens Health. (2020) 20:9. doi: 10.1186/s12905-019-0878-z, 31952500 PMC6967084

[37] Salmon VE Hay-Smith EJC Jarvie R Dean S Terry R Frawley H . Implementing pelvic floor muscle training in women's childbearing years: a critical interpretive synthesis of individual, professional, and service issues. Neurourol Urodyn. (2020) 39:863–70. doi: 10.1002/nau.24256, 31845393 PMC7079154

[38] Fernandes ACNL Palacios-Ceña D Hay-Smith J Pena CC Sidou MF de Alencar AL . Women report sustained benefits from attending group-based education about pelvic floor muscles: a longitudinal qualitative study. J Physiother. (2021) 67:210–6. doi: 10.1016/j.jphys.2021.06.010, 34147398

[39] Gard AJM Lavallee D. Perceptions of a ‘pelvic-floor friendly’ group exercise class in women with urinary incontinence. Appl Sci. (2025) 15:2705. doi: 10.3390/app15052705

[40] Vlaev I King D Dolan P Darzi A. The theory and practice of "nudging": changing health behaviors. Public Adm Rev. (2016) 76:550–61. doi: 10.1111/puar.12564

[41] Jaffar A Mohd Sidik S Foo CN Muhammad NA Abdul Manaf R Suhaili N. Preliminary effectiveness of mHealth app-based pelvic floor muscle training among pregnant women to improve their exercise adherence: a pilot randomised control trial. Int J Environ Res Public Health. (2022) 19:2332. doi: 10.3390/ijerph19042332 35206520 PMC8872112

[42] Dufour S Fedorkow D Kun J Deng SX Fang Q. Exploring the impact of a Mobile health solution for postpartum pelvic floor muscle training: pilot randomized controlled feasibility study. JMIR Mhealth Uhealth. (2019) 7:e12587. doi: 10.2196/12587, 31298221 PMC6657451

[43] Marteau TM Ogilvie D Roland M Suhrcke M Kelly MP. Judging nudging: can nudging improve population health? BMJ. (2011) 342:d228. doi: 10.1136/bmj.d228, 21266441

[44] Brewer NT Chapman GB Rothman AJ Leask J Kempe A. Increasing vaccination: putting psychological science into action. Psychol Sci Public Interest. (2017) 18:149–207. doi: 10.1177/1529100618760521, 29611455

[45] Patel MS Volpp KG Asch DA. Nudge units to improve the delivery of health care. N Engl J Med. (2018) 378:214–6. doi: 10.1056/NEJMp1712984, 29342387 PMC6143141

[46] Harper RC Sheppard S Miller E Stewart C Clark CJ. DryByChristmas: a patient and public involvement study on women's engagement with humorous pelvic floor muscle training digital nudges on social media. Health Expect. (2024) 27:e14033. doi: 10.1111/hex.14033, 38556833 PMC10982603

[47] Burgio KL Cunningham SD Newman DK Low LK Nodora J Lipman TH . Preferences for public health messaging related to bladder health in adolescent and adult women. J Womens Health (Larchmt). (2023) 32:1120–35. doi: 10.1089/jwh.2022.0463 37610853 PMC10541935

[48] Leask CF Sandlund M Skelton DA Altenburg TM Cardon G Chinapaw MJM . Framework, principles and recommendations for utilising participatory methodologies in the co-creation and evaluation of public health interventions. Res Involv Engagem. (2019) 5:2. doi: 10.1186/s40900-018-0136-9 30652027 PMC6327557

[49] Xu P Wang X Guo P Zhang W Mao M Feng S. The effectiveness of eHealth interventions on female pelvic floor dysfunction: a systematic review and meta-analysis. Int Urogynecol J. (2022) 33:3325–54. doi: 10.1007/s00192-022-05222-5 35616695 PMC9135393

[50] Geoffrion R Badowski S Gong M Mann G Tilak M Koenig N . Pelvic floor health index: initial validation of a practical postpartum tool for busy clinicians. Can Fam Physician. (2023) 69:e229–35. doi: 10.46747/cfp.6911e229, 37963795 PMC10645455

[51] Amit G Girshovitz I Marcus K Zhang Y Pathak J Bar V . Estimation of postpartum depression risk from electronic health records using machine learning. BMC Pregnancy Childbirth. (2021) 21:630. doi: 10.1186/s12884-021-04087-8 34535116 PMC8447665

[52] Woodley SJ Moller B Clark AR Bussey MD Sangelaji B Perry M . Digital technologies for women's pelvic floor muscle training to manage urinary incontinence across their life course: scoping review. JMIR Mhealth Uhealth. (2023) 11:e44929. doi: 10.2196/44929, 37405818 PMC10357376

[53] Weinstein MM Dunivan G Guaderrama NM Richter HE. Digital therapeutic device for urinary incontinence: a randomized controlled trial. Obstet Gynecol. (2022) 139:606–15. doi: 10.1097/AOG.0000000000004725, 35271539 PMC8936159

[54] Harvie HS Sung VW Neuwahl SJ Honeycutt AA Meyer I Chermansky CJ . Cost-effectiveness of behavioral and pelvic floor muscle therapy combined with midurethral sling surgery vs surgery alone among women with mixed urinary incontinence: results of the effects of surgical treatment enhanced with exercise for mixed urinary incontinence randomized trial. Am J Obstet Gynecol. (2021) 225:651.e1–651.e26. doi: 10.1016/j.ajog.2021.06.099, 34242627 PMC8633051

[55] Cacciari LP Kouakou CR Poder TG Vale L Morin M Mayrand MH . Group-based pelvic floor muscle training is a more cost-effective approach to treat urinary incontinence in older women: economic analysis of a randomised trial. J Physiother. (2022) 68:191–6. doi: 10.1016/j.jphys.2022.06.001, 35753969

[56] World Health Organization In: World Health Organization, editor. WHO guideline recommendations on digital interventions for health system strengthening. Geneva: (2019) 31162915

[57] Tulenko K Møgedal S Afzal MM Frymus D Oshin A Pate M . Community health workers for universal health-care coverage: from fragmentation to synergy. Bull World Health Organ. (2013) 91:847–52. doi: 10.2471/BLT.13.118745, 24347709 PMC3853952

[58] Chen CCG Cox JT Yuan C Thomaier L Dutta S. Knowledge of pelvic floor disorders in women seeking primary care: a cross-sectional study. BMC Fam Pract. (2019) 20:70. doi: 10.1186/s12875-019-0958-z, 31122187 PMC6533649

[59] Lorig KR Holman H. Self-management education: history, definition, outcomes, and mechanisms. Ann Behav Med. (2003) 26:1–7. doi: 10.1207/S15324796ABM2601_01, 12867348

[60] Asklund I Nyström E Sjöström M Umefjord G Stenlund H Samuelsson E. Mobile app for treatment of stress urinary incontinence: a randomized controlled trial. Neurourol Urodyn. (2017) 36:1369–76. doi: 10.1002/nau.23116, 27611958

[61] Nie XF Rong L Yue SW Redding SR Ouyang YQ Zhang Q. Efficacy of community-based pelvic floor muscle training to improve pelvic floor dysfunction in Chinese Perimenopausal women: a randomized controlled trial. J Community Health Nurs. (2021) 38:48–58. doi: 10.1080/07370016.2020.1869416, 33682549

[62] Bo K Frawley HC Haylen BT Abramov Y Almeida FG Berghmans B . An international Urogynecological association (IUGA)/international continence society (ICS) joint report on the terminology for the conservative and nonpharmacological management of female pelvic floor dysfunction. Neurourol Urodyn. (2017) 36:221–44. doi: 10.1002/nau.23107, 27918122

[63] Bazi T Takahashi S Ismail S Bø K Ruiz-Zapata AM Duckett J . Prevention of pelvic floor disorders: international urogynecological association research and development committee opinion. Int Urogynecol J. (2016) 27:1785–95. doi: 10.1007/s00192-016-2993-9 26971276

